# Pseudoaneurysmal bone cyst secondary to primary hyperparathyroidism mimicking early infection following hip arthroplasty: A case report

**DOI:** 10.1177/2050313X261441736

**Published:** 2026-05-04

**Authors:** Lennard M. Wurm, Boris Möbius, Lukas Neuhaus, Wolfgang Ertel, Justyna Reinke

**Affiliations:** 1 Department of Traumatology and Reconstructive Surgery, Charité – Universitätsmedizin Berlin, Freie Universität Berlin, Humboldt-Universität zu Berlin and Berlin Institute of Health, Germany; 2 Medical Faculty of Heinrich-Heine University and University Hospital Düsseldorf, Germany

**Keywords:** primary hyperparathyroidism, brown tumor, aneurysmal bone cyst, periprosthetic joint infection, total hip arthroplasty

## Abstract

Pseudoaneurysmal bone cysts are rare complications of advanced primary hyperparathyroidism, mimicking malignant lesions or postoperative infections, particularly when accompanied by wound drainage and elevated inflammatory markers. A 49-year-old man underwent cemented total hip arthroplasty for a pathological femoral neck fracture. Persistent postoperative bloody-serous discharge and elevated infection markers such as C-reactive protein suggested early periprosthetic joint infection, prompting two surgical revisions. During both revision procedures, multiple deep tissue samples were obtained and sent for aerobic and anaerobic cultures with extended incubation as well as histopathological analysis. All microbiological cultures remained negative. Histopathology revealed a giant cell-rich, hemorrhagic, fibrous lesion consistent with a pseudoaneurysmal bone cyst due to undiagnosed primary hyperparathyroidism. Timely surgical debridement, microbiological sampling, and empiric antibiotics ensured patient safety while the metabolic etiology was being established. Biochemical tests confirmed hypercalcemia and elevated parathyroid hormone from a parathyroid adenoma. Primary hyperparathyroidism-induced osteoclastic activity can produce hemorrhagic cystic lesions resembling infection or malignancy. Meticulous surgical exploration, repeated microbiological sampling, and histopathological analysis excluded infection and malignancy, while definitive management via parathyroidectomy prevents further skeletal complications. This case emphasizes structured diagnostics and interdisciplinary orthopedic-endocrine collaboration for managing rare primary hyperparathyroidism complications effectively and underlines that persistent postoperative drainage with repeatedly negative cultures should prompt metabolic evaluation.

## Introduction

Primary hyperparathyroidism (PHPT) results from excessive parathyroid hormone (PTH) secretion, most frequently due to a solitary adenoma, causing chronic hypercalcemia and heightened osteoclastic bone resorption.^1^ Although improved biochemical screening has made severe PHPT manifestations rare, advanced skeletal changes still occur and can present as the generalized bone disease osteitis fibrosa cystica. Within this spectrum, localized and hemorrhagic lesions are frequently referred to as pseudoaneurysmal bone cysts (PBC, historically or clinically often termed brown tumors). These lesions manifest as expansile cysts,^2,3^ that may mimic malignancies (e.g. giant cell tumors, telangiectatic osteosarcomas) or even aneurysmal bone cysts (ABCs).^4,5^

In certain cases, particularly after orthopedic procedures, the presentation can be further complicated by signs typically suggestive of infection, such as persistent wound drainage and elevated inflammatory markers.^6^ Distinguishing between acute infection and a hemorrhagic, metabolically driven bone lesion is imperative to avoid unnecessary prolonged antibiotic therapy or misdirected oncologic treatments.

We report the case of a 49-year-old man who initially underwent a total hip arthroplasty (THA) due to an atraumatic pathological fracture suspected to be malignant. Postoperatively, persistent bloody-serous wound discharge and elevated infection markers mimicked an early joint infection, prompting urgent surgical revisions. Ultimately, histological and biochemical workup revealed a Pseudoaneurysmal bone cyst (PSA) secondary to undiagnosed PHPT. We emphasize how methodical surgical and diagnostic measures, combined with interdisciplinary collaboration, led to definitive diagnosis and appropriate management.

## Case description

**Figure 1.**
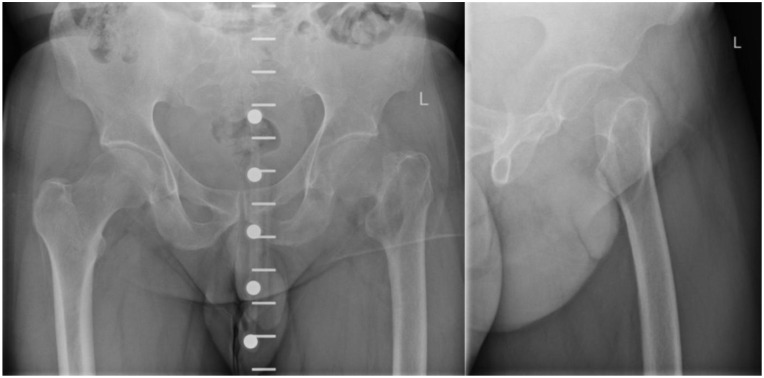
Initial preoperative anteroposterior and additive axial radiograph of the left hip demonstrating an acute, atraumatic pathological fracture of the femoral neck.

A 49-year-old male patient underwent a cemented THA on February 19, 2025, to address an atraumatic pathological fracture of the left femoral neck (Figure 1). Initial preoperative radiographs revealed an expansile osteolytic lesion at the fracture site. Due to the acute nature of the fracture requiring prompt surgical stabilization, a preoperative bone biopsy was not performed and routine preoperative laboratory testing unfortunately did not include a serum calcium assessment. During the index procedure, the operating team noted an expansile, hemorrhagic, and cystic bone lesion, initially raising concern for a possible sarcoma. Nonetheless, no definitive malignancy was confirmed intraoperatively, and the hip arthroplasty proceeded uneventfully. Over the following weeks, the patient began experiencing persistent bloody-serous drainage from the surgical wound.

On March 1, 2025, he was readmitted with ongoing wound secretion and elevated infection markers such as C-reactive protein (CRP). Although he remained hemodynamically stable, a periprosthetic joint infection (PJI) could not be excluded. Given the possibility of an early postoperative infection, we performed a revision surgery on March 3, 2025, which included extensive debridement of the soft tissue and partial removal of the prosthetic components (polyethylene inlay, femoral head, and femoral stem). During the revision, multiple deep periprosthetic tissue samples (at least three distinct sites) were collected for aerobic and anaerobic microbiological cultures as well as histopathological analysis. Postoperatively, empirical antibiotic therapy with clindamycin and ampicillin–sulbactam was initiated while awaiting final results.

Despite these measures, bloody-serous wound drainage persisted, prompting a second revision and open debridement on March 7, 2025. Again, samples were taken for culture and intraoperative inspection raised the suspicion that the lesion might not be purely infectious. Once the pathological results returned, they revealed the presence of a giant cell-rich, hemorrhagic, fibrous lesion consistent with a PSA. Simultaneously, microbiological cultures remained negative, which effectively ruled out a bacterial cause. Once all microbiological cultures remained sterile after 14 days and the diagnosis of a PBC was confirmed, the empirical antibiotic therapy was immediately discontinued.

Further investigations uncovered markedly elevated serum calcium and PTH levels, culminating in a diagnosis of PHPT. Parathyroid scintigraphy subsequently confirmed a single hyperfunctioning parathyroid adenoma as the underlying etiology. With this metabolic and endocrine explanation, the persistent wound discharge was best explained by the excessive vascularity and friability associated with the PSA rather than an infection. The patient’s infection markers gradually normalized, leading to cessation of antibiotic therapy. He responded well to wound care and was mobilized under partial weight-bearing restrictions at the bedside.

**Figure 2.**
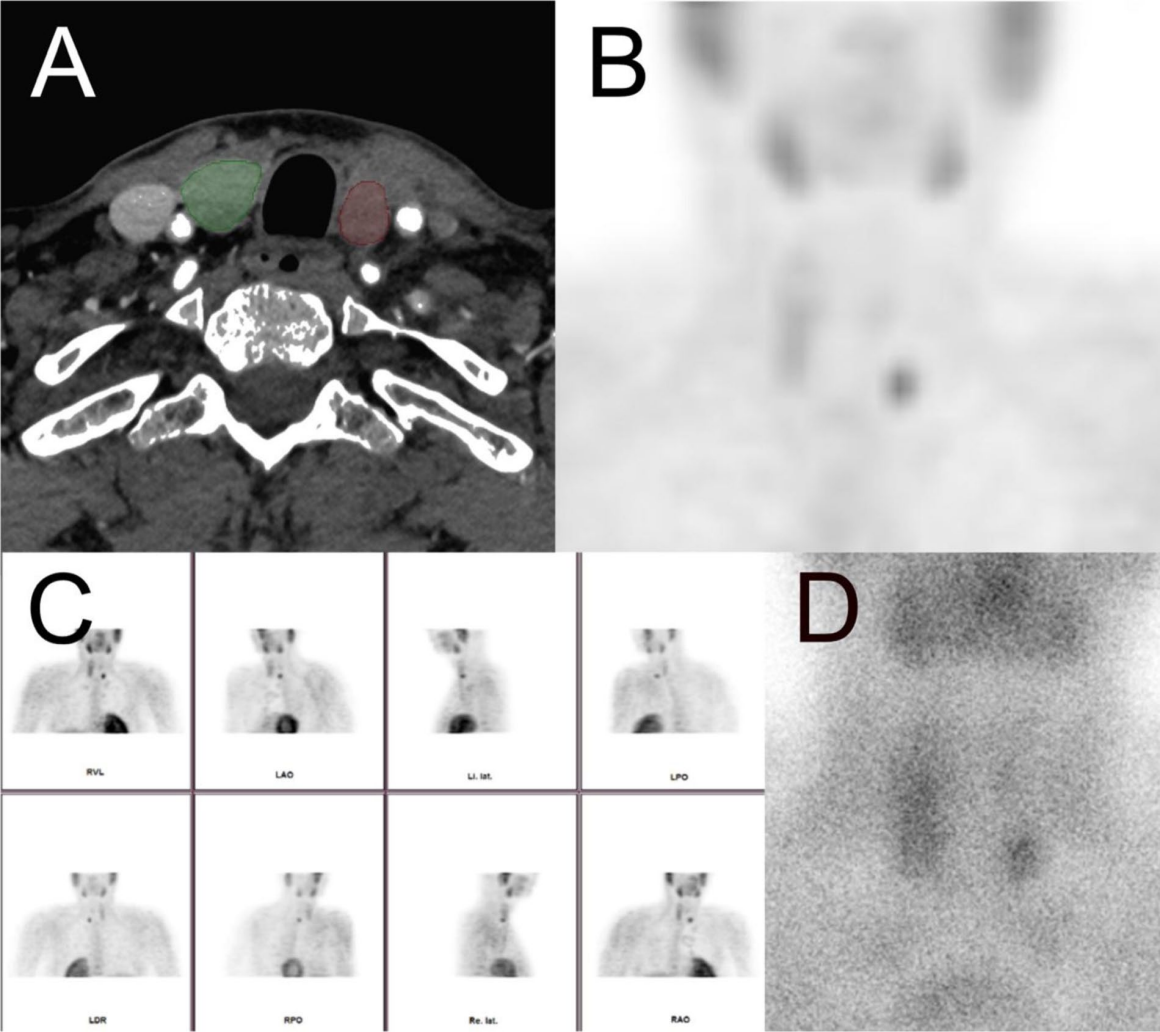
Imaging findings in the patient with primary hyperparathyroidism due to a parathyroid adenoma. (a) Axial contrast-enhanced Computed Tomography (CT) scan of the neck demonstrates a well-circumscribed, hypodense lesion posterior to the left thyroid lobe (marked in red), consistent with a parathyroid adenoma. The right thyroid lobe is shown for comparison (green). (b) Coronal PET image showing increased tracer uptake at the site of the left parathyroid adenoma, indicating metabolic activity. (c) Multiplanar scintigraphy images using a Sestamibi scan demonstrate focal tracer retention corresponding to the left inferior parathyroid gland, consistent with an adenoma. (d) Planar image from Single-Photon Emission Computed Tomography (SPECT) or early static scintigraphy phase further confirms focal activity in the left parathyroid region, supporting the diagnosis of a metabolically active parathyroid adenoma. These imaging findings are typical for primary hyperparathyroidism due to a functioning parathyroid adenoma.

**Figure 3.**
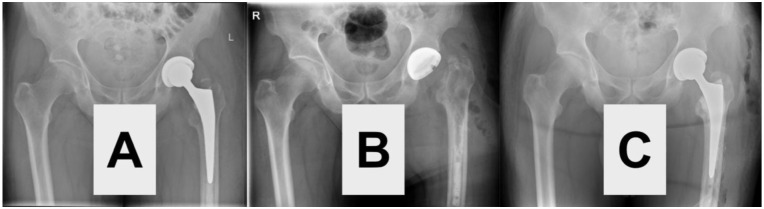
Radiographic findings of a pseudoaneurysmal bone cyst in the setting of primary hyperparathyroidism. (a) Pelvic radiograph shows a total hip arthroplasty on the left side preoperatively, while the left femoral head exhibits a multiloculated, expansile osteolytic lesion with cortical thinning and septation, radiographically consistent with a brown tumor or pseudoaneurysmal bone cyst. (b) Anteroposterior view of the pelvis without prosthesis, demonstrating the same osteolytic lesion of the proximal right femur with ballooning of the cortex and well-defined margins, suggesting a benign but aggressive lesion. (c) Follow-up radiograph post-treatment (after parathyroidectomy and correction of hyperparathyroidism) shows reossification and partial consolidation of the cystic bone lesion, consistent with healing of a brown tumor. These findings are consistent with a brown tumor (osteitis fibrosa cystica), a reactive bone lesion secondary to excess PTH in primary hyperparathyroidism. The lesion’s pseudoaneurysmal appearance can mimic neoplastic or vascular pathology but is reversible upon correction of the underlying endocrine disorder. PTH: parathyroid hormone.

**Figure 4.**
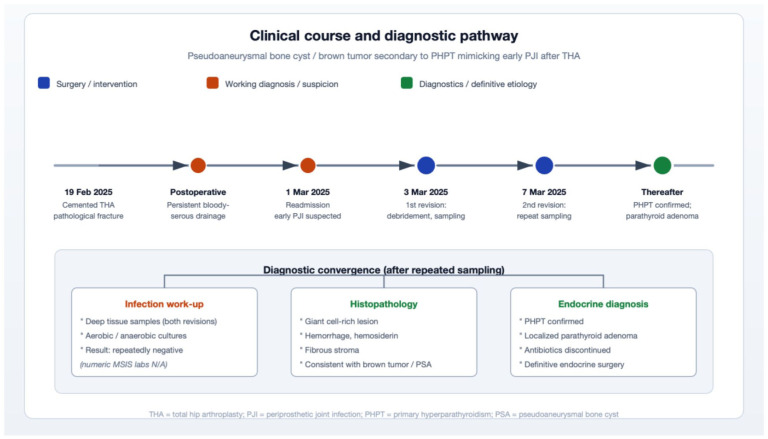
Clinical course and diagnostic pathway. Schematic overview illustrating the chronological sequence of clinical events from the index total hip arthroplasty (February 19, 2025) through the two revision procedures (March 3 and March 7, 2025) to the definitive endocrine diagnosis and surgical treatment. The lower panel summarizes the three converging diagnostic pillars: infection work-up (repeatedly negative cultures), histopathology (giant cell-rich hemorrhagic fibrous lesion consistent with brown tumor), and endocrine evaluation (confirmed PHPT with parathyroid adenoma). THA: total hip arthroplasty; PJI: periprosthetic joint infection; PHPT: primary hyperparathyroidism.

Because definitive resolution of the underlying pathology requires targeted treatment of PHPT, the patient was transferred to an endocrine surgery department for parathyroidectomy (Figure 2). This procedure corrected his hypercalcemia, halted the progression of osteitis fibrosa cystica and fostered regression of PSA over time before reimplantation of the prosthesis (Figure 3). The patient recovered well so that we were able to restore the patient’s mobility promptly by reimplanting a total hip endoprosthesis and subsequent rehabilitation. The complete clinical course and diagnostic pathway are illustrated in Figure 4.

## Discussion

This case exemplifies the complexity of diagnosing and managing a rare skeletal manifestation of PHPT that initially mimicked an early postoperative infection. Despite progressive wound drainage and elevated inflammatory markers, a clinical picture highly suggestive of PJI, the structured approach of surgical debridement, repeated tissue sampling and histopathological investigation was instrumental in reaching the correct diagnosis. The negative microbiological workup and the identification of a benign giant cell-rich lesion not only ruled out infection but also raised the index of suspicion for an endocrine cause.

PSA in the context of PHPT stem from chronically elevated PTH, which drives significant osteoclastic activity.^7^ Repeated microfractures and local hemorrhages give rise to blood-filled cavities separated by fibrous septa lined with osteoclastic giant cells. These lesions can appear both radiologically and grossly similar to malignant processes such as telangiectatic osteosarcoma, or benign conditions like giant cell tumors and ABCs.^8^ In parallel, the presence of wound seepage that was dark and serosanguinous contributed to the initial suspicion of an infectious or malignant entity. Histologically, brown tumors are typically characterized by multinucleated osteoclast-like giant cells embedded in a fibroblastic stroma, with prominent intralesional hemorrhage and hemosiderin deposition reflecting repeated cycles of bleeding and resorption.^8^ These features, which were consistent with the findings in our patient, are key to distinguishing brown tumors from true neoplastic processes and infectious etiologies.

Fortunately, the management strategy adopted in our unit balanced vigilance for infection with an openness to alternative diagnoses.^9^ By obtaining multiple deep tissue biopsies and maintaining a broad differential, we ensured that no diagnosis was prematurely dismissed. The histological features consistent with PSA and concurrent laboratory findings, particularly severe hypercalcemia, ultimately confirmed PHPT as the root cause. This illustrates a central tenet of orthopedic oncology and infection control: Correlation of clinical, laboratory, and pathological data is indispensable for accurately distinguishing among infection, malignancy, and unusual metabolic lesions.

A particularly important diagnostic pitfall highlighted by this case is the clinical overlap between metabolic bone disease and PJI. Persistent postoperative drainage with elevated inflammatory markers naturally triggers an infection-focused algorithm; however, when repeated cultures remain negative, the differential diagnosis should be explicitly broadened to include metabolic and endocrine etiologies.

Crucially, this case highlights that the routine assessment of serum calcium levels should be a mandatory component of the preoperative laboratory checklist for any patient presenting with an atraumatic or suspected pathological fracture, as early identification of hypercalcemia can prevent misdiagnosis and alter the surgical approach.

Furthermore, hyperparathyroidism-related bone fragility can significantly impact the arthroplasty setting. Chronic PTH excess not only predisposes to pathological fractures but may also compromise implant fixation and perioperative wound healing due to altered bone metabolism and increased vascularity of affected tissue. Clinicians should therefore maintain a heightened awareness of metabolic bone disease when confronted with unexpected perioperative complications (Supplemental Material).

Once PSA are identified, definitive treatment of PHPT through parathyroidectomy remains essential for preventing further skeletal complications. Correcting the endocrine imbalance typically leads to lesion stabilization and sometimes complete or partial regression of the cystic cavities. Although local surgical interventions, such as curettage or fixation, may be needed to manage symptomatic or structurally significant PSAs, the long-term outcome hinges primarily on controlling the underlying hyperparathyroidism.^10^ Our patient’s course, which included thorough orthopedic revisions followed by referral for definitive endocrine surgery, underscores the benefits of interdisciplinary collaboration in such a complex scenario.

## Conclusion

A PSA secondary to PHPT may mimic various pathological processes, including infection and malignancy, especially in the early postoperative setting. This case demonstrates how a diligent, stepwise approach, surgical debridement, systematic sampling, and comprehensive endocrine evaluation correctly elucidated the metabolic origin of the patient’s bone lesion and wound drainage. Definitive parathyroid surgery is expected to stabilize and potentially reverse the skeletal changes. Early recognition of brown tumors in patients with hypercalcemia can avert unnecessary antimicrobial or oncologic therapies, reinforce the need for interdisciplinary cooperation and ultimately lead to favorable clinical outcomes.

## Supplemental Material

sj-pdf-1-sco-10.1177_2050313X261441736 – Supplemental material for Pseudoaneurysmal bone cyst secondary to primary hyperparathyroidism mimicking early infection following hip arthroplasty: A case report Supplemental material, sj-pdf-1-sco-10.1177_2050313X261441736 for Pseudoaneurysmal bone cyst secondary to primary hyperparathyroidism mimicking early infection following hip arthroplasty: A case report by Lennard M. Wurm, Boris Möbius, Lukas Neuhaus, Wolfgang Ertel and Justyna Reinke in SAGE Open Medical Case Reports

## References

[1] Meng J Aboznadah WM Pusztaszeri M , et al. Primary hyperparathyroidism due to a giant parathyroid adenoma presenting with pathological fractures and multiple brown tumors. Endocrinol Diabetes Metab Case Rep 2024; 2024(4): e240054. 10.1530/EDM-24-0054 PMC11737434 39700332

[2] Nasser ML Medawar S Younan T , et al. Osteitis fibrosa cystica mimicking bone tumor, a case report. BMC Musculoskelet Disord 2021; 22: 479. 34034731 10.1186/s12891-021-04374-7 PMC8152144

[3] Basaran Y Ince S Alagoz E , et al. An unusual presentation of primary hyperparathyroidism: multiple brown tumors and coexisting thyroid carcinoma. Rev Esp Med Nucl Imagen Mol 2016; 35: 321–324. 27036887 10.1016/j.remn.2016.02.005

[4] Zhou Z Shi Y Li C , et al. Primary hyperparathyroidism-induced brown tumors caused by parathyroid carcinoma: a case report and literature review. J Int Med Res 2022; 50: 03000605221123668.

[5] Sivakumar SP Vasudeva N Perumal R , et al. Primary hyperparathyroidism mimicking skeletal metastasis – a diagnostic dilemma. J Orthop Case Rep 2024; 14(1): 103–108. 10.13107/jocr.2024.v14.i01.4158 PMC10823813 38292108

[6] Xu W Qu Y Shi W , et al. Multiple bone brown tumor secondary to primary hyperparathyroidism: a case report and literature review. Gland Surg 2019; 8: 810–816. 32042691 10.21037/gs.2019.11.14 PMC6989901

[7] Dinoi E Prete A Sardella C , et al. The challenge of the differential diagnosis between brown tumors and metastases in parathyroid carcinoma: a case report. Front Endocrinol 2024; 15: 1414896. 10.3389/fendo.2024.1414896 PMC11527640 39493781

[8] Zhong Y Huang Y Luo J , et al. Misdiagnosis of brown tumour caused by primary hyperparathyroidism: a case report with literature review. BMC Endocr Disord 2022; 22: 66. 35287634 10.1186/s12902-022-00971-2 PMC8919606

[9] Wurm LM Neuhaus L Aspargur G , et al. Pseudohypoxic brain swelling following cerebrospinal fluid leakage: a case report on rapid identification and multidisciplinary management. J Surg Case Rep 2024; 2024: rjae520. 10.1093/jscr/rjae520 PMC11333088 39161424

[10] Panagopoulos A Tatani I Kourea HP , et al. Osteolytic lesions (brown tumors) of primary hyperparathyroidism misdiagnosed as multifocal giant cell tumor of the distal ulna and radius: a case report. J Med Case Rep 2018; 12: 176. 29936913 10.1186/s13256-018-1723-y PMC6016128

